# Addressing Female Genital Mutilation/Cutting (FGM/C) in the Era of Clitoral Reconstruction: Plastic Surgery

**DOI:** 10.1007/s11930-018-0147-4

**Published:** 2018-04-27

**Authors:** Hannes Sigurjonsson, Malin Jordal

**Affiliations:** 1 Department of Plastic and Reconstructive Surgery, Karolinska University Hospital, 171 76 Stockholm, Sweden; 2 Department of Medicine, Karolinska Institute, Stockholm, Sweden; 3 Center for Gender Research, Uppsala University, Uppsala, Sweden

**Keywords:** Clitoris, Clitoral reconstruction, Multi-disciplinary approach, Female genital mutilation, Female genital cutting, reconstructive plastic surgery

## Abstract

**Purpose of the Review:**

The aim of this review is to give an overview of the recent evidence on clitoral reconstruction and other relevant reconstructive plastic surgery measures after female genital mutilation/cutting (FGM/C).

**Recent Findings:**

Recent publications present refinements and modifications of the surgical technique of clitoral reconstruction along with reconstruction of the labia majora and clitoral hood. Novel approaches with reposition of the clitoral nerve, anchoring of the labia majora, fat grafting, and full thickness mucosa grafts have been introduced. The current evidence on outcomes of clitoral reconstruction shows potential benefits. However, there is a risk of postoperative complications and a negative outcome. Experts in the field advocate for a multidisciplinary approach including psychosexual counseling and health education with or without subsequent clitoral reconstructive surgery.

**Summary:**

The evolution of reconstructive treatment for women with FGM/C is expanding, however at a slow rate. The scarcity of evidence on clitoral reconstruction halters availability of clinical guidelines and consensus regarding best practice. Clitoral reconstruction should be provided by multidisciplinary referral centers in a research setting with long-term follow-up on outcomes of postoperative morbidity and possible benefits.

## Introduction

Reconstructive surgery to improve function and aesthetics of the genitalia of women living with consequences of female genital mutilation/cutting (FGM/C) is increasing in demand [1•]. It includes reconstruction of the clitoris and labia, defibulation, and removal of cysts, neuromas, and scar tissue. Clitoral reconstruction, which this review focuses on, has been proposed to be a safe and efficient surgical procedure which can restore identity, reduce pain, and improve sexual function [2–6]. However, thorough evidence on motives, expectations, and outcome is scarce. Available studies have been criticized for methodological limitations, low number of patients, and short follow-up time [7, 8]. Furthermore, because of heterogeneity of the outcome measures, the existing studies are hard to compare [9••, 10••]. The purpose of this review is to give an overview of recent evidence on clitoral reconstruction, modifications, and refinements of the technique and other reconstructive procedures aimed to improve the lives of women with FGM/C.

The surgical outcome of clitoral reconstruction is reported in a few published case series with short-term follow-up, ranging from 1 to 12 months postoperatively [2–5], as well as in a recent comprehensive systematic review [10••]. Postoperative complications ranged from 5 to 15%. The most frequent complications were hematoma, infection, and wound dehiscence. A visible neo-glans was observed in 77.5% (range 70–100%) at follow-up. Improvement in sexual function was observed, with less vulvar pain in 43% of the women on average (range 8–77%). Clitoral function with regard to sensitivity and sexual pleasure was improved in 63% (range 61–89%) of the patients, sexual desire by 51% (range 47–56%) [2–5]. One study reported that coital bleeding resolved for 15% and vaginal pain for 26% of the women [11]. Psychosexual outcome was found to improve after clitoral reconstruction in a study by Abramowicz et al. [2] where 88–96% (*n* = 19) of the women had improved their body image, aesthetic appearance, and psychological well-being 6–12 months postoperatively.

Limited evidence exists on negative effects of clitoral reconstruction. In the study by Foldés et al. (*n* = 866), a decrease in sexual pleasure was experienced by 2.3% of the women. Also 8.2% of the women experienced decreased desire and 2.0% suffered from postoperative genital pain that was not experienced preoperatively. A high number of women, 22.6% (12 out of 53), who had regular orgasm preoperatively experienced dysfunctional orgasmic function postoperatively [3].

Anatomical and imaging studies of the clitoris have clarified the anatomy and terminology of the external female genitalia [12]. Different types of FGM/C result in a wide scope of genital trauma and functional impairment. The severity of damage does not always correlate with the degree of sexual impairment or pain as seen in a recent magnetic resonance imaging study. This study compared the volumes of clitoral tissue between cut and uncut women in a non-aroused state, and showed that cut women had slightly significantly smaller volume of the clitoris plus vestibular bulbs, but no difference on only the clitoris, clitoral glans, and body [13•].

## Clitoral Reconstruction

The procedure of clitoral reconstruction was first described by Thabet and Thabet [6] and Foldés et al. [3]. As described by these authors, the incision in the skin is made over the clitoral shaft stump. Dissection is proceeded cranially to the clitoral stump, preserving the dorsal neurovascular bundle. Close to the pubic bone, the suspensory ligament is transected to a degree that it allows sufficient mobilization to an appropriate anatomical position. A neo-glans is created and sutured to the skin with projection after resection of residual scar and fibrotic tissue. Concomitant reconstructive surgery (e.g., defibulation, removal of cysts) is done if necessary. Although the procedure can be done solely under local anesthesia with or without intravenous sedation, it has been recommended to operate under general anesthesia. The use of local anesthesia and adequate postoperative analgesia is important to prevent the patient from re-experiencing the pain and trauma from initial FGM/C or other violence (e.g., sexual assault, rape) [3, 14•]. One case of postoperative relapse of posttraumatic stress disorder has been documented [14•].

### Motives and Expectations of Clitoral Reconstruction

FGM/C and clitoral reconstruction are complex issues with medical, social, gendered, and psychosexual implications [9••]. Body image and gender identity play a determinant role in health, well-being, sexuality [15], in all complex and multifactorial aspects for research in the context of clitoral reconstruction. To optimize care for women who have undergone FGM/C, such research could contribute to guidelines for patient selection and who could benefit from surgery.

There is an increasing body of evidence available which explores motives and expectations of women seeking clitoral reconstruction. The reports state that women disclose a desire to improve sexual function, wanting to recover one’s identity, and hoping to reduce vulvar pain as the strongest motives for undergoing clitoral reconstruction [2, 3, 11, 16–18]. An unpublished study from Sweden shows that women opting for clitoral reconstruction do so as they want to symbolically reclaim what they perceived as forcefully taken from them during the act of cutting, aesthetically repair the visibly “damaged” genitalia, improve their sexual function, and eliminate physical pain. These factors were all intertwined, indicating that the reasons for seeking surgery are often multiple and complex [19].

### Recent Studies on New Reconstruction Techniques After FGM/C

A recent publication from Germany presents a novel approach in reconstructing both the clitoris as well as the labia majora and clitoral hood [20]. The publication does not describe the surgical techniques in detail, merely presents them as a novelty. The author presents a method he calls “neurotizing and molding of the clitoral stump,” where the clitoral nerves are re-integrated to the distal clitoral stump. A pedicled fasciocutaneous flap is raised from the inner thigh and tunneled to the vulva to reconstruct the labia majora as an aesthetical unit after FGM/C type III (with infibulation) [21]. The flap, designed oval in shape, is based on a perforator from the anterior obturator artery. The clitoral hood is reconstructed with a local skin flap, cranially based, in an omega shape, called the omega dome flap [20, 22].

A preliminary report from Chang et al. [23] of three women undergoing clitoral reconstruction after WHO type II FGM/C describes a novel technique of anchoring the labia majora to the pubic bone to reduce the risk of labial fusion. Furthermore, the authors describe that instead of excising a small part of the skin over the clitoral stump, they incise and spare all the skin, scarred or not, for resurfacing of the labia majora. Instead of releasing the clitoral stump from the pubic bone, as has been described by previous authors [3, 6], only the surrounding tissue was released and labia anchored besides the clitoris. Postoperative wound care of the clitoris was managed with sutured petroleum dressing with daily moistening by the patient with local anesthetic cream and antibiotic ointment until the clitoris re-epithelialized [24].

A recent study from Spain reported the outcome of clitoral reconstruction using vaginal mucosal grafts on 32 women affected by FGM/C. The surgical technique included excision of fibrotic scar tissue, dissection of the suspensory ligament for clitoral stump mobilization, and fixation. The surgeons then harvested a 3–4-cm-long and 2-cm-wide mucosa graft from the posterior vagina that was transplanted on to the reconstructed clitoris up to the labia majora. A urinary catheter was left in place for 5 days while the patient was on bed rest. Loss to follow-up were five patients. The results showed successful graft survival in all cases (*n* = 27) but two cases of partial necrosis of the graft that healed by secondary intention. Patients answered two validated questionnaires preoperatively and 6 months postoperatively, the Female Sexual Function Index and the Female Genital Self-Image Index. There was a significant increase in the scores of both questionnaires postoperatively. No intraoperative or postoperative complications were noted [25•].

### Other Reconstructive Surgeries After FGM/C

Reconstructive surgery after FGM/C, besides clitoral reconstruction, includes defibulation [26, 27], reconstruction of the labia and removal of cysts [28–31], and excision of neuromas [32, 33]. These are performed separately or in conjunction with each other to tackle a spectrum of different problems caused by FGM/C. A recent meta-analysis reported a favorable effect of defibulation in general, with respect to cesarean section and perineal tears at birth, as well as an indication of improved sexual function [10••]. A study with a 6-month follow-up on patients operated with excision of cysts has shown a positive outcome when the excision was performed in conjunction with reconstructive surgery of the clitoris, but worsening of sexual function if done solely [6]. A case series published on the effects of excision of neuromas has reported good results [33]. The authors followed up seven women with clitoral pain operated with clitoral reconstruction. Only one of the women was diagnosed with neuroma preoperatively but histological exam revealed neuroma in all seven cases. All women experienced resolution of pain and there were no recurrences of neuroma [33]. A case series of excision of keloids as a complication of FGM/C was recently published. The authors describe clitoral masses of 27 women operated upon. Pathology confirmed all excised masses as keloids and no short-term recurrences (after 1 year) [34].

## Discussion

In some settings, women affected by FGM/C are increasingly seeking clitoral reconstruction to reduce pain, improve sexual function, and regain identity [17•]. Before a new surgical procedure is widely implemented, sufficient evidence on safety and efficacy should be established [9••, 21, 35]. This has not been the case when it comes to clitoral reconstruction [10••]. Therefore, it is questionable to advocate for a widespread use of the technique [23] which has been discouraged by several experts in the field pointing out the infancy of the evidence of this procedure [36, 37].

Limitations in the existing studies of outcome of clitoral reconstruction are the use of non-validated scales, a relatively short follow-up time (≤ 1 year), and a significant loss to follow-up [2–5]. Furthermore, few studies are able to report on the outcomes, including the outcomes of psychosexual effects of the surgery in a contextualized and nuanced way. Also, the high rate of reduced orgasmic function found in one study is alarming [3]. This negative outcome could be explained by nerve damage during the surgery; however, this is yet unclear. Another important research gap is the lack of evidence on non-surgical strategies to improve self-identity and psychosexual function [9••, 35].

The scientific evidence on the effect of defibulation and removal of cysts is also limited, even if the facilitation of sexual intercourse, pregnancy, childbirth, voiding, and menstruation with defibulation or removal of large cysts is obvious [10••]. Formation of keloids is a known complication after FGM/C, and a short-term (12 months) follow-up study of keloid excision has shown good results [34]. However, keloids tend to be recurrent [38]; thus, long-term follow-up studies are warranted, as are follow-up studies on keloid formation after clitoral reconstruction. Careful surgical technique and tissue manipulation, choice of sutures that trigger low immune reaction like absorbable biopolymer monofilaments, minimizing tension on suture lines, and precise wound border alignment can all reduce the formation of hypertrophic scars and keloids. In addition, there is a variety of adjuvant approaches to reduce keloids or their chances of recurrence, including intralesional corticosteroids and radiation brachytherapy, which have proved successful management choices for keloids [39, 40].

### Multidisciplinary Approach

Sexuality is multifactorial and FGM/C can have a major negative impact, both physically and psychologically, on sexual function [41]. However, not all women subjected to FGM/C experience sexual problems. Some studies indicate that older women that grow up in FGM/C-practicing countries, where being cut is considered normal, have better sexual function compared to women, often younger, who grow up in settings where FGM/C is regarded as mutilation [3, 41]. Women with FGM/C, who are often migrants, also have a higher prevalence of having experienced physical and psychiatric trauma (often resulting from war, rape, and forced marriage) than the majority population in western host countries [42]. Experts in the field have advocated for a multidisciplinary approach by referral centers including psychosexual counseling along with health education with or without subsequent reconstructive surgery for women with FGM/C [1•, 16]. The goal of the multidisciplinary treatment is often to improve psychosexual well-being, treat psychological and sexological comorbidities, and empower women to make an informed decision on treatment choices, which may include clitoral reconstruction, based on medical, psychological, sexological, and cultural aspects [1•, 43, 44].

With regard to surgical reconstruction, the cooperation of different specialties includes the efforts of plastic surgeons, gynecologists, and urologists. Bringing together aspects from the field of experts can enhance and improve the outcome as seen in for example gender reassignment surgery [45]. Gender reassignment surgeons often have extensive knowledge in genital anatomy and well-developed surgical skills with regard to the clitoris. One example is skills in metoidioplasty which is a widely used technique for phallus reconstruction in transsexual men. This technique uses the hypertrophied clitoris, achieved by cross-hormone therapy, to reconstruct a microphallus. To elongate the clitoris, the clitoral hood is lifted and the suspensory ligament dissected and urethral lengthening done with local flaps from adjacent mucosa and labia minora [46]. This underlines the importance of sharing knowledge and skills to the benefit of the relatively new patient group of women who seek reconstructive surgery after FGM/C.

### Experiences from Sweden

Migration from FGM/C-practicing countries resulted in an estimated 38,000 FGM/C-affected women and girls living in Sweden [47]. This combined with the low overall population numbers in Sweden means that Sweden has one of the highest prevalence figures of women living with FGM/C in Europe. Almost half of the cut women in Sweden are from Somalia where WHO type III FGM/C is common [47]. Because immigration to Sweden from FGM/C-practicing countries is likely to continue, the Swedish health care system must be prepared to receive patients with FGM/C-related health care needs. Clitoral reconstruction was introduced in Sweden in early 2015 and is covered by the National Health Insurance. The team responsible for the patient group decided that the surgery should be carried out in the context of multidisciplinary cooperation, and that the women opting for surgery should meet a psychotherapist and sexologist both pre- and postoperatively. The team includes specialists in gynecology, reconstructive plastic surgery, psychiatry, psychotherapy, sexology, and midwives. Regular multidisciplinary meetings are held to discuss patients and their needs. The patients are planned to be followed up short and long term, at 1 and 3 years post-operatively, and assessed with regard to motives and expectations, psychosexual well-being, complications, and outcome of the surgery.

**Fig. 1:**
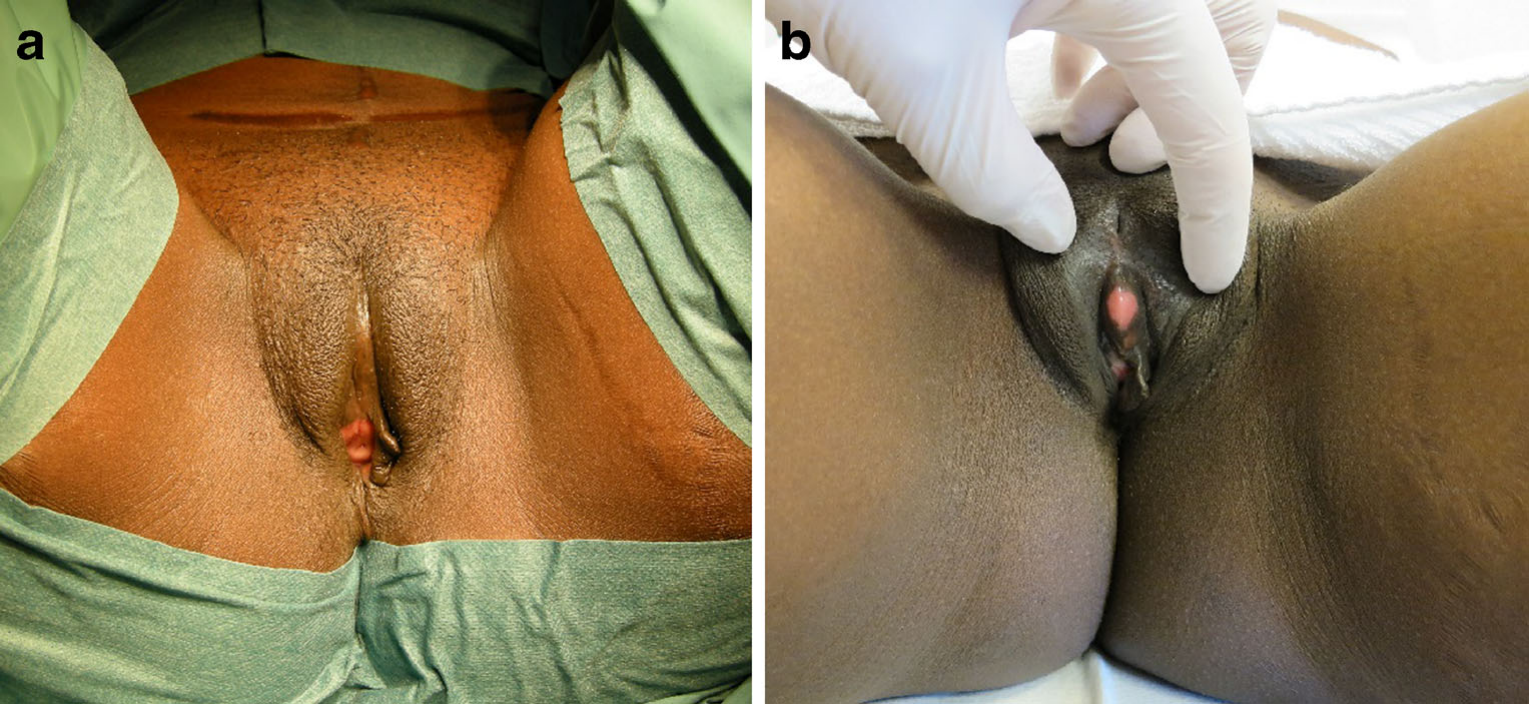
Preoperative status of a 33-year-old woman with WHO type II FGM/C (**a**). Postoperative at 12 months after clitoral reconstruction and a full thickness mucosal graft from the lateral vaginal wall (**b**)

The surgical protocol for clitoral reconstruction involves general anesthesia. Long-acting local anesthesia as well as postoperative topical and oral analgesia is used. The surgical technique described by Foldès [3] is performed, often with modifications: (1) Full thickness mucosal graft from the lateral vaginal wall is transplanted to the clitoral neo-glans to promote faster healing (Fig. 1); (2) fat grafting of the labia majora and above the clitoris is done in case of severe tissue loss and scarring after FGM/C; (3) local skin flaps are used to reconstruct the clitoral hood if there is sufficient adjacent unscarred skin. Mean operation time is 45 min and length of stay 24 h.

### Future Research

Future research should focus on multicenter collaboration and identifying validated scales, or create new ones, to improve homogeneity of outcome measures.

Engorgement of the clitoral tissue is seen as a prerequisite for orgasm due to the stimuli of the clitoris [12] and, when impaired, can have a significant impact on sexual function. This has been shown in women with atherosclerosis and metabolic syndrome with color Doppler ultrasound [12, 48, 49]. The remaining clitoral tissue of women subjected to FGM/C is often embedded in scar tissue, retracted to the pubic bone [3]. The theory behind clitoral reconstruction is to relieve the clitoral stump from the scar bed, make it more available, and facilitate engorgement and erection for improved orgasmic function. The anatomical study by Abdulcadir et al. [13•] found a slight difference between the total volumes of clitoral and bulbar tissue between cut and uncut women, but not between the volumes of the clitoris alone. To provide information on clitoral erectile insufficiency of women with FGM/C, imaging studies (e.g., ultrasound Doppler, MRI) of both objective and subjective clitoral function of women with FGM/C are warranted. It would be interesting to compare volume differences and blood flow to the clitoris in both non-aroused and aroused state, as well as compare to uncut women and between non-reconstructed and reconstructed women with FGM/C in the context of subjective functionality.

Furthermore, basic plastic surgery treatment principals have been established for vulvovaginal reconstruction for congenital malformations or tissue loss after cancer surgery and trauma. However, these techniques have not yet been described thoroughly for treatment of the FGM/C population of women. The techniques could include free and pedicled flaps and less invasive techniques more likely to be applicable for FGM/C women, like local flaps, fat grafting, full thickness skin, or mucosa grafts. Non-FGM/C-related reconstruction of the labia majora with fat grafting has been reported for both reconstructive [50] and aesthetic [51] purposes. As FGM/C can leave the labia majora scarred [13•] or partially or totally remove them, this procedure could propose a possible beneficial treatment of vulvar pain both from labia majora and clitoral area as for increased protection of sensitive structures of the vulva, vaginal introitus, urethral meatus, and clitoral area after loss of tissue.

In clitoral reconstruction, the neo-glans is left to heal by secondary intention. Full thickness skin grafts and mucosal skin grafts are widely used in reconstructive plastic surgery and could be beneficial for faster healing of the neo-glans, reducing infection rates, postoperative pain, and give a more aesthetically pleasing appearance and texture [25•]. Sensitivity measurements, of vibratory and tactile thresholds, of the clitoris of uncut women exist [52] as well as for transsexual women with reconstructed neo-clitoris [53]. To date, there are no sensitivity measurements of women with FGM/C published. A study of the sensitivity thresholds of women with FGM/C before and after clitoral reconstruction correlated with psychosexual outcome would shine more light on this.

## Conclusions

New applications of reconstructive techniques might benefit some women with FGM/C. However, more research is needed to establish these as is the case for clitoral reconstruction in general. Fortunately, not all cut women need reconstructive surgery and as evidence is still lacking, patients requesting clitoral reconstruction should be informed that current available data on safety and outcomes of clitoral reconstruction is still limited. Also, clitoral reconstruction should be provided by multidisciplinary referral centers in a study setting with long-term follow-up on outcomes of safety, morbidity, and possible postoperative effects on pain, psychosexual well-being, and body image.

## References

[1] De Schrijver L, Leye E, Merckx M (2016). A multidisciplinary approach to clitoral reconstruction after female genital mutilation: the crucial role of counselling. Eur J Contracept Reprod Health Care.

[2] Abramowicz S, Oden S, Dietrich G, Marpeau L, Resch B (2016). Anatomic, functional and identity results after clitoris transposition. J Gynecol Obstet Biol Reprod.

[3] Foldes P, Cuzin B, Andro A (2012). Reconstructive surgery after female genital mutilation: a prospective cohort study. Lancet (Lond, Engl).

[4] Foldes P, Louis-Sylvestre C (2006). Results of surgical clitoral repair after ritual excision: 453 cases. Gynecol Obstet Fertil.

[5] Ouedraogo CM, Madzou S, Toure B, Ouedraogo A, Ouedraogo S, Lankoande J (2013). Practice of reconstructive plastic surgery of the clitoris after genital mutilation in Burkina Faso. Report of 94 cases. Ann Chir Plast Esthet.

[6] Thabet SM, Thabet AS (2003). Defective sexuality and female circumcision: the cause and the possible management. J Obstet Gynaecol Res.

[7] Creighton S, Bewley S, Liao LM (2012). Reconstructive surgery after female genital mutilation. Lancet (Lond, Engl).

[8] Zhang GY, Li QF, Cai JL, Fu XB, Gao WY (2012). Reconstructive surgery after female genital mutilation. Lancet (Lond, Engl).

[9] Abdulcadir J, Rodriguez MI, Say L (2015). A systematic review of the evidence on clitoral reconstruction after female genital mutilation/cutting. Int J Gynaecol Obstet.

[10] •• Berg RC, Taraldsen S, Said MA, Sorbye IK, Vangen S. The effectiveness of surgical interventions for women with FGM/C: a systematic review. BJOG Int J Obstet Gynaecol. 2018;125(3):278–87. 10.1111/1471-0528.14839. **A recent systematic review on effectiveness of clitoral reconstruction.** 10.1111/1471-0528.14839 28755440

[11] Merckelbagh HM, Nicolas MN, Piketty MP, Benifla JL (2015). Assessment of a multidisciplinary care for 169 excised women with an initial reconstructive surgery project. Gynecol Obstet Fertil.

[12] Puppo V (2013). Anatomy and physiology of the clitoris, vestibular bulbs, and labia minora with a review of the female orgasm and the prevention of female sexual dysfunction. Clin Anat (New York, NY).

[13] Abdulcadir J, Botsikas D, Bolmont M, Bilancioni A, Djema DA, Bianchi Demicheli F (2016). Sexual anatomy and function in women with and without genital mutilation: a cross-sectional study. J Sex Med.

[14] Abdulcadir J, Bianchi Demicheli F, Willame A, Recordon N, Petignat P (2017). Posttraumatic stress disorder relapse and clitoral reconstruction after female genital mutilation. Obstet Gynecol.

[15] Parker R (2009). Sexuality, culture and society: shifting paradigms in sexuality research. Cult Health Sex.

[16] Abdulcadir J, Rodriguez MI, Petignat P, Say L (2015). Clitoral reconstruction after female genital mutilation/cutting: case studies. J Sex Med.

[17] Berg RC, Taraldsen S, Said MA, Sorbye IK, Vangen S (2017). Reasons for and experiences with surgical interventions for female genital mutilation/cutting (FGM/C): a systematic review. J Sex Med.

[18] Vital M, de Visme S, Hanf M, Philippe HJ, Winer N, Wylomanski S (2016). Using the Female Sexual Function Index (FSFI) to evaluate sexual function in women with genital mutilation undergoing surgical reconstruction: a pilot prospective study. Eur J Obstet Gynecol Reprod Biol.

[19] Sigurjonsson H, Jordal M, Lundgren TK. Reconstructive surgery after female genital mutilation. Int J Gynaecol Obstet. 2015;131 (Suppl 5): E318–9.

[20] O'Dey DM (2017). Complex vulvar reconstruction following female genital mutilation/cutting. Urologe A.

[21] WHO. WHO guidelines on the management of complications from female genital mutilation 2016 [16.2.2018]. Available from: http://www.who.int.proxy.kib.ki.se/reproductivehealth/topics/fgm/management-health-complications-fgm/en/. 27359024

[22] O'Dey DM, Bozkurt A, Pallua N (2010). The anterior obturator artery perforator (aOAP) flap: surgical anatomy and application of a method for vulvar reconstruction. Gynecol Oncol.

[23] Chang CS, Low DW, Percec I (2017). Female genital mutilation reconstruction: a preliminary report. Aesthet Surg J.

[24] Chang CS, Low DW, Percec I (2017). Response to letters regarding “female genital mutilation reconstruction: a preliminary report”. Aesthet Surg J.

[25] Manero I, Labanca T (2018). Clitoral reconstruction using a vaginal graft after female genital mutilation. Obstet Gynecol.

[26] Nour NM, Michels KB, Bryant AE (2006). Defibulation to treat female genital cutting: effect on symptoms and sexual function. Obstet Gynecol.

[27] Krause E, Brandner S, Mueller MD, Kuhn A (2011). Out of Eastern Africa: defibulation and sexual function in woman with female genital mutilation. J Sex Med.

[28] Amu OC, Udeh EI, Ugochukwu AI, Madu C, Nzegwu MA (2012). A case of vulval swelling secondary to female circumcision posing a diagnostic dilemma. Int J Surg Case Rep.

[29] Aziem-Abdallah-Ali A, Mohammed AA, Mohammed Ali AK (2011). Large inclusion cyst complicating female genital mutilation. Clin Pract.

[30] Kaur-Desai T, Makris A. Massive epidermal vulval cyst: an unusual late complication of female genital mutilation. BMJ Case Rep. 2017; 10.1136/bcr-2017-220335. 10.1136/bcr-2017-220335 PMC5614244 28814588

[31] Victoria-Martinez AM, Cubells-Sanchez L, Martinez-Leborans L, Sanchez-Carazo JL, de Miquel VA (2016). Vulvar epidermal inclusion cyst as a long-term complication of female genital mutilation. Indian J Dermatol.

[32] Abdulcadir J, Pusztaszeri M, Vilarino R, Dubuisson JB, Vlastos AT (2012). Clitoral neuroma after female genital mutilation/cutting: a rare but possible event. J Sex Med.

[33] Abdulcadir J, Tille JC, Petignat P (2017). Management of painful clitoral neuroma after female genital mutilation/cutting. Reprod Health.

[34] Birge O, Akbas M, Ozbey EG, Adiyeke M (2016). Clitoral keloids after female genital mutilation/cutting. Turk J Obstet Gynecol.

[35] Gynaecologists. RCoOa. Female genital mutilation and its management. Green-top Guideline No. 53 www.rcog.org.uk2015 [cited 2018 16.2.2018]. Available from: https://www.rcog.org.uk/en/guidelines-research-services/guidelines/gtg53/.

[36] Atkinson HG, Bowers M, Mishori R, Ottenheimer D (2017). Comments on “female genital mutilation reconstruction: a preliminary report”. Aesthet Surg J.

[37] Abdulcadir J, Abdulcadir O, Caillet M, Catania L, Cuzin B, Essen B (2017). Clitoral surgery after female genital mutilation/cutting. Aesthet Surg J.

[38] Trace AP, Enos CW, Mantel A, Harvey VM (2016). Keloids and hypertrophic scars: a spectrum of clinical challenges. Am J Clin Dermatol.

[39] Arno AI, Gauglitz GG, Barret JP, Jeschke MG (2014). Up-to-date approach to manage keloids and hypertrophic scars: a useful guide. Burns.

[40] Edriss A, Mesták J (2005). Management of keloid and hypertrophic scars. Ann Burns Fire Disasters.

[41] Catania L, Abdulcadir O, Puppo V, Verde JB, Abdulcadir J, Abdulcadir D (2007). Pleasure and orgasm in women with female genital mutilation/cutting (FGM/C). J Sex Med.

[42] Akinsulure-Smith AM, Chu T (2017). Exploring female genital cutting among survivors of torture. J Immigr Minor Health.

[43] Smith H, Stein K (2017). Psychological and counselling interventions for female genital mutilation. Int J Gynaecol Obstet.

[44] Antonetti Ndiaye E, Fall S, Beltran L (2015). Benefits of multidisciplinary care for excised women. J Gynecol Obstet Biol Reprod.

[45] Colebunders B, Brondeel S, D'Arpa S, Hoebeke P, Monstrey S (2017). An update on the surgical treatment for transgender patients. Sex Med Rev.

[46] Hage JJ (1996). Metaidoioplasty: an alternative phalloplasty technique in transsexuals. Plast Reconstr Surg.

[47] Socialstyrelsen. Kvinnor och flickor som kan ha varit utsatta för könsstympning. En uppskattning av antalet. www.socialstyrelsen.se: Socialstyrelsen, 2015.

[48] Park K, Goldstein I, Andry C, Siroky MB, Krane RJ, Azadzoi KM (1997). Vasculogenic female sexual dysfunction: the hemodynamic basis for vaginal engorgement insufficiency and clitoral erectile insufficiency. Int J Impot Res.

[49] Maseroli E, Fanni E, Cipriani S, Scavello I, Pampaloni F, Battaglia C, Fambrini M, Mannucci E, Jannini EA, Maggi M, Vignozzi L (2016). Cardiometabolic risk and female sexuality: focus on clitoral vascular resistance. J Sex Med.

[50] Vogt PM, Herold C, Rennekampff HO (2011). Autologous fat transplantation for labia majora reconstruction. Aesthet Plast Surg.

[51] Salgado CJ, Tang JC, Desrosiers AE (2012). Use of dermal fat graft for augmentation of the labia majora. J Plast Reconstr Aesthet Surg.

[52] Cordeau D, Belanger M, Beaulieu-Prevost D, Courtois F (2014). The assessment of sensory detection thresholds on the perineum and breast compared with control body sites. J Sex Med.

[53] Sigurjonsson H, Mollermark C, Rinder J, Farnebo F, Lundgren TK (2017). Long-term sensitivity and patient-reported functionality of the neoclitoris after gender reassignment surgery. J Sex Med.

